# Why is vascular dysfunction an important target for reducing alternate bearing in *Coffea arabica*?

**DOI:** 10.3389/fpls.2026.1803340

**Published:** 2026-04-27

**Authors:** Thalita de Carvalho Gonçalves, Roberto Tarazi

**Affiliations:** Universidade Federal do Rio de Janeiro (UFRJ), Duque de Caxias, RJ, Brazil

**Keywords:** alternate bearing (AB), biennial bearing, coffee, vascular tissue, xylem and phloem

## Introduction

Coffee is one of the most valuable crops in the world (FAO, 2025), and it provides a livelihood for more than 125 million people worldwide (Sachs et al., 2019). The genus *Coffea* belongs to the Rubiaceae family and comprises 131 described species (Ramalho et al., 2025). The most cultivated coffee varieties descend from two wild *Coffea* species: *Coffea arabica* L. (Arabica coffee) and *C. canephora* Pierre ex A. Froehner. (Robusta coffee) (Vanden Abeele et al., 2021). *Coffea arabica* is an allotetraploid hybrid (4n = 4x = 44) of *C. eugenioides* S. Moore and *C. canephora* and contributes to approximately 60% of world coffee production (Scalabrin et al., 2024). *Coffea arabica* and *C*. *canephora* have similar management requirements; however, *C. arabica* is more sensitive to biotic and abiotic stress and has a higher alternate bearing intensity (DaMatta et al., 2025).

Alternate (biennial) bearing (AB) is a reproductive phenomenon in which episodic floweringtriggers the reallocation of carbon, nutrients, and hormonal signals, manifesting as cycles of high-yield (“on”) years followed by low-yield (“off”) years. AB occurs across many crops of global socioeconomic importance, including apple, avocado, coffee, mango, olive, and pistachio (Goldschmidt and Sadka, 2021; Monselise and Goldschmidt, 1982). Despite its clear implications for production stability, AB has received limited attention in discussions of socio-ecological resilience (Garcia et al., 2024). In *Coffea arabica*, AB constitutes a major constraint on farmer livelihoods because income and market access depend on consistent harvests in both quantity and quality (Garcia et al., 2025). Most research efforts have focused on agronomic measures to increase productivity and climate resilience, but few studies have systematically evaluated how these interventions directly influence AB (Garcia and Orians, 2022).

In fruit trees, AB is a complex, multi-scale phenomenon framed by different theoretical models, such as the Resource Budget Model (RBM) as applied by Garcia and Orians (2022) and the Reproductive/Vegetative biomass ratio model (REP/VEG) articulated by Goldschmidt (2022). The RBM suggests that increased resources lead to excessive fruiting, deplete whole−plant resources, and reduce reproduction the next season. The REP/VEG model emphasizes local allocation balance, organ-level signaling, and developmental thresholds that determine bud fate. In our view, these frameworks are complementary: RBM provides a quantitative energetic backbone, while REP/VEG supplies mechanistic detail on allocation, architecture, and hormonal control. Integrating both, yields clearer hypotheses for experiments and more targeted management strategies. This integrated view explains several empirical observations: (1) fruit thinning or defruiting can restore next-year flowering by both conserving reserves and lowering local REP/VEG (León-Burgos et al., 2024); (2) fertilization can mitigate but not always eliminate alternate bearing because it addresses resource pools but may not reset local allocation or signaling (Zheng et al., 2019); (3) Plant growth regulators (PGRs) and pruning can shift bud fate rapidly by altering local REP/VEG and hormonal balances even when whole-plant reserves remain low (Jan et al., 2022).

From a physiological and molecular perspective, AB arises from cross-regulatory pathways that modulate a core set of floral integrators, restricting flowering to favorable environmental and developmental conditions (Khan et al., 2025). Four major genetic pathways control flowering in *Arabidopsis thaliana* by integrating external signals and the plant’s internal state to trigger the switch from vegetative growth to flowering (Blázquez et al., 2001).

Although models describing cross-regulated pathways and resource reallocation in alternate bearing exist, an important question persists: Why do current agricultural management strategies not consistently mitigate alternate bearing in *Coffea arabica*?

## Solving the challenge

The probable answer is that agricultural management strategies do not target the plant vascular system, which conducts all the plant’s resources and signals. A holistic understanding of vascular function and its regulation is indispensable for unraveling and managing alternate bearing in *C*. *arabica* and other perennial crops.

## Theoretical background

Damage to vascular tissues—xylem and phloem—through cavitation, embolism, blockage, or pathogen attack disrupts the integrated transport of water, nutrients, carbohydrates, and signaling molecules and thereby alters the physiological processes that determine flowering, fruit set, and bud development (Zhang and Brodribb, 2017; Konrad et al., 2018; Qaderi et al., 2019). In *C. arabica*, xylem cavitation occurs when water stress or high evaporative demand increases xylem sap tension, drawing air into the conduits and forming emboli that interrupt the continuous water column and reduce hydraulic conductivity (Nardini et al., 2014). Xylem anatomy strongly determines the likelihood and severity of cavitation: wider vessels increase hydraulic efficiency but raise vulnerability to embolism, whereas narrower, more reinforced conduits trade transport capacity for safety (Max et al., 2023).

Cavitation limits water supply to leaves and reproductive organs, provoking stomatal closure, reducing photosynthetic carbon gain, and causing leaf wilting, abscission and ultimately lower growth and yield; recovery after rewatering is often incomplete or delayed because embolized vessels may require active refilling processes that depend on carbohydrate availability and intact phloem function Tausend et al., 2000; Carréra et al., 2023). Empirical work on coffee documents substantial genotypic variation in hydraulic traits and cavitation vulnerability, so that cultivar choice, rooting depth, and water-use strategy materially influence susceptibility and recovery under intermittent drought (Nardini et al., 2014; dos Santos et al., 2025; Tausend et al., 2000).

Phloem dysfunction compounds these effects by impairing the long-distance transport of photosynthates, hormones, and signaling molecules from source leaves to sink organs such as developing fruits, buds, and roots (De Schepper et al., 2013). Sieve tubes and companion cells mediate sucrose movement and the distribution of regulatory compounds that coordinate growth, storage, and developmental transitions; phloem blockage can arise from pathogen invasion, phloem-feeding insects, mechanical injury, or stress-induced callose deposition and collapse of sieve elements (Konrad et al., 2018). When phloem transport is compromised, sinks receive insufficient carbohydrates and hormonal cues, resulting in reduced fruit size and quality, inhibited bud development, leaf chlorosis, and, in severe cases, necrosis and dieback Nardini et al., 2011). Because the phloem actively contributes to refilling embolized xylem conduits, phloem impairment can both limit reserve remobilization and slow hydraulic recovery, creating a coupled vascular failure that undermines the plant’s capacity to restore source strength after a heavy crop year (De Schepper et al., 2013).

## Our rationale

**Figure 1:**
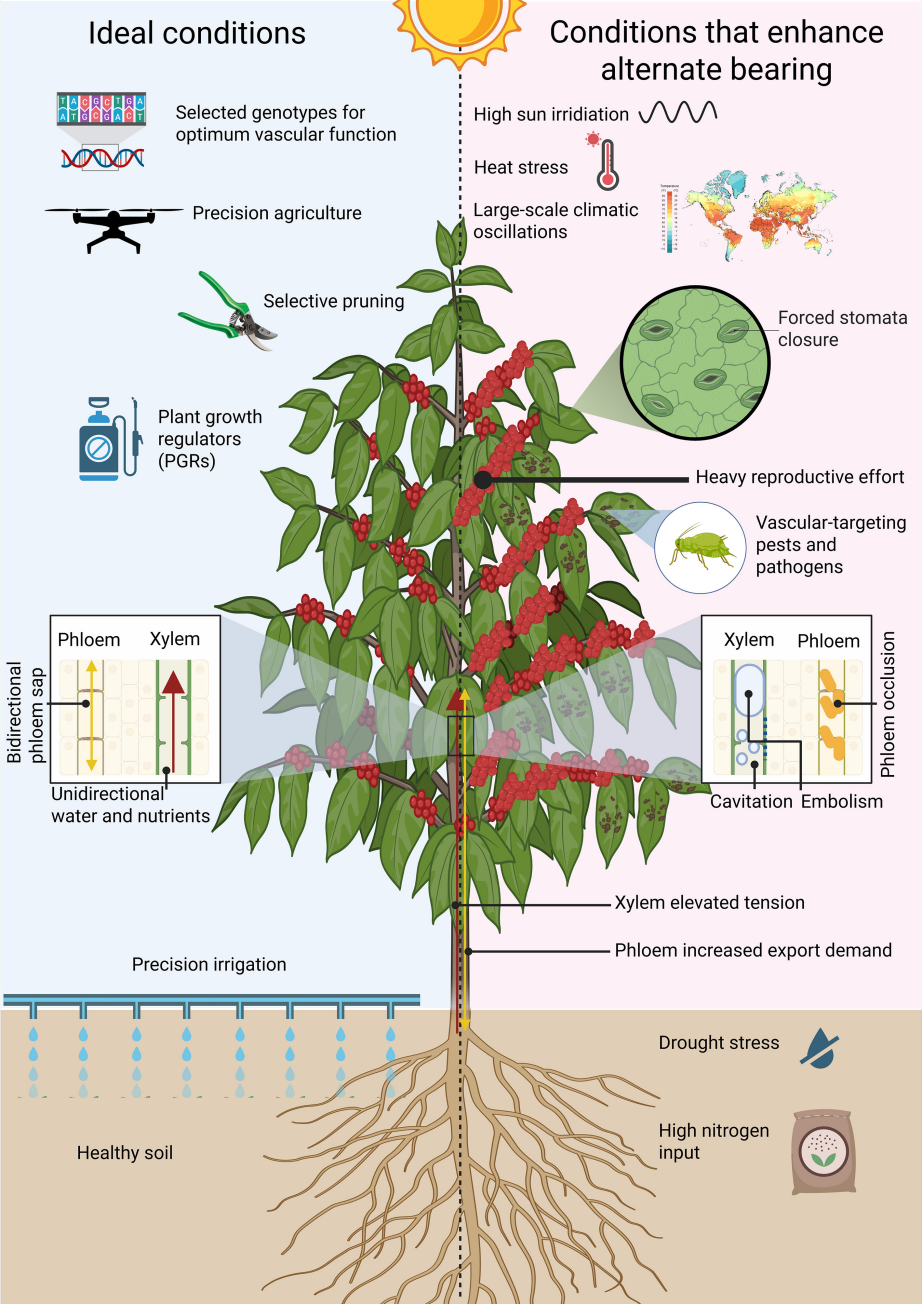
Contrasting conditions that influence vascular function in coffee plants, highlighting how idealenvironments support steady growth and fruiting while stressors drive alternate bearing. Under ideal conditions, optimal genotypes, combined with practices such as precision agriculture, selective pruning, and precision irrigation, maintain healthy soil and promote efficient transport of water and nutrients, enabling balanced development. In contrast, factors like intense sunlight, heat, climatic oscillations, drought, high nitrogen input, pests, pathogens, and heavy reproductive effort increase xylem tension and phloem export demand, disrupting vascular flow through mechanisms such as cavitation, embolism, and phloem occlusion, ultimately leading to irregular fruit production. Created in BioRender. Tarazi, R. (2026) https://BioRender.com/xh41out.

We propose that coupled xylem–phloem dysfunction provides a mechanistic link between genotype, agronomic management, climatic variability, biotic agents, and alternate bearing (AB) in *Coffea arabica* (**Figure 1**). Direct evidence tying AB to vascular failure remains limited, but studies of AB species offer indirect support and reveal physiological and ecological patterns consistent with this model.

In our framework, genotype strongly mediates the relationship between xylem–phloem dysfunction and AB. Under resource-rich conditions (for example, high nitrogen and strong irradiance), high-yielding genotypes produce large fruit loads in “on” years. These heavy fruit loads create dominant local sinks, sharply increasing phloem export demand and xylem water requirements. Genotypes that combine high yield potential with anatomical traits such as wider xylem vessels (which require more water and are more vulnerable to cavitation) or thinner phloem (which are more prone to occlusion and reduced assimilate flow) are especially predisposed to AB. Elevated phloem demand, together with increased xylem tension, can lead to persistent vascular impairment, limiting carbohydrate and water delivery to developing tissues and thereby reducing yields in subsequent seasons.

Empirical observations are consistent with this interpretation. For example, *C. arabica* varieties Paraíso MG1, Araponga MG1, and Catuaí Vermelho IAC 15 differ in vascular traits and AB intensity (Santos et al., 2022). Paraíso MG1 is high yielding, has wider but fewer xylem vessels, lower water-use efficiency, greater drought susceptibility, and stronger AB than Araponga MG1 and Catuaí Vermelho IAC 15, which have narrower vessels, higher vessel counts, better water-use efficiency, and lower AB. Although the work by Santos et al. (2022) does not directly demonstrate vascular failure as the cause of AB, their results align with our model. Similarly, the drought-adapted, high-yielding variety MGS Turmalina shows more xylem vessels, thicker phloem, and lower AB than Catuaí Vermelho IAC 15 and IAC 99 (Oliveira et al., 2025), suggesting that genotypes with less xylem–phloem dysfunction exhibit lower AB intensity. From a breeding perspective, these patterns imply that it is possible to select for both high yield and reduced AB by targeting hydraulic architecture and water-use efficiency to minimize vascular dysfunction, an idea aligned with Merga et al. (2023) ‘s findings on high-yielding and lower alternate-bearing genotypes.

To mitigate vascular dysfunction, breeding should prioritize genotypes that combine anatomical and physiological traits conferring vascular resilience. Traits of interest include narrower xylem vessels, thicker phloem, larger hydraulic safety margins, greater nonstructural carbohydrate (NSC) reserves, and faster phloem recovery after stress. Additional desirable traits include small leaves with high vein density (Nardini et al., 2014) and resistance to vascular-targeting pests and pathogens (for example, phloem-feeding insects and stem cankers) that directly damage conductive tissues or trigger defensive occlusion responses, thereby reducing transport capacity (Venzon, 2021). Establishing standardized, high-throughput protocols for measuring these traits will enable selection of genotypes with a lower propensity for AB and provide a mechanistic basis for genotype-specific management.

Because new genotypes take many years to reach growers and wholesale replanting is often cost-prohibitive, near-term emphasis should be on affordable agronomic practices that preserve vascular integrity and reduce AB incidence. Priority measures include irrigation, selective pruning, and targeted application of plant growth regulators.

Irrigation is likely the most important management tool for coffee because targeted water supply stabilizes flowering and fruit set (Masarirambi et al., 2009), maximizes yield, preserves bean quality (Barros et al., 1997), and maintains production under variable climatic conditions (DaMatta et al., 2006). Large-scale climatic oscillations (e.g., El Niño–Southern Oscillation and the Madden–Julian Oscillation) modulate drought and heat stress, amplifying hydraulic strain and increasing the probability of vascular failure during critical phenological windows (Sarvina et al., 2021); such synchronous stress events can intensify AB at landscape scales.

Precision irrigation stabilizes soil–plant water status, preventing recurrent water stress and xylem embolism by avoiding extreme negative stem water potentials that drive cavitation (Anjum et al., 2023). By matching water timing and volume to crop demand, it reduces water-potential excursions, maintains safer midday stem and leaf water status, and promotes root development and soil moisture stability, thereby lowering stomatal extremes, dampening xylem tension swings, and enhancing hydraulic resilience (Bracken et al., 2023). Field studies show that irrigation can increase *C. arabica* yields even in years of otherwise low production (Sakai et al., 2015), supporting an indirect link among water deficit, vascular dysfunction, and AB.

Selective pruning performs two complementary functions in managing vascular-related productivity decline in *Coffea arabica*. First, when coupled xylem–phloem dysfunction, canopy function is already compromised, and irrigation cannot restore productivity; selective pruning acts as a reinvigoration treatment that promotes recovery of productive capacity (Fernandes et al., 2012). Second, when applied prophylactically, selective pruning reduces the risk of vascular failure by altering canopy architecture and lowering whole-plant hydraulic demand (Pinkard and Beadle, 2000; Gokavi et al., 2021). Targeted pruning of orthotropic (vertical) branches further contributes to hydraulic resilience by stimulating the development of vigorous plagiotropic (horizontal) branches. Orthotropic branches typically possess larger vessel diameters, whereas plagiotropic branches have narrower, more numerous vessels (Carréra et al., 2023); reducing the proportion of orthotropic growth therefore decreases susceptibility to hydraulic conductivity loss and may reduce alternate bearing.

Exogenous application of plant growth regulators, such as triazole retardants, produces compact canopies with reduced transpiration demand and an increased root: shoot ratio (Desta and Amare, 2021). In practice, paclobutrazol (PBZ) currently offers the most consistent, crop-specific evidence for shifting assimilate partitioning and improving drought resilience in *C*. *arabica*, but its use requires careful dose and timing control, as well as integration with irrigation and nutrient management (Ribeiro et al., 2017). PBZ enhances drought resilience, thereby reducing susceptibility to hydraulic conductivity loss and potentially mitigating alternate bearing.

In conclusion, a mechanistic understanding of xylem–phloem structure and function, coupled with targeted breeding and agronomic practices that preserve vascular integrity, provides a practical strategy to mitigate alternate bearing in *Coffea arabica*.
